# Supplementary material for: Exploring the Structurally Conserved Regions and Functional Significance in Bacterial N-Terminal Nucleophile (Ntn) Amide-Hydrolases
Source: Int J Mol Sci. 2024 Jun 21;25(13):6850. doi: 10.3390/ijms25136850 (PMC11241749; doi:10.3390/ijms25136850)
Supplement: Supplementary file 1 [file ijms-25-06850-s001.zip › ijms-3031591-supplementary.pdf]

Table S1. The table presents a comprehensive list of 83 protein structures PDB codes, Merops class, corresponding proteins, PDB structure groups, species, and protein lengths for various enzymes. The enzymes include penicillin G acylase precursor, cephalosporin acylase precursor, D-succinylase, and acyl-homoserine lactone acylase. The Merops class categorizes them into specific enzyme classes. Each entry includes detailed information about the protein, its structure group, and its length. The proteins originate from various bacterial species such as *Escherichia coli*, *Providencia rettgeri*, *Kluyvera citrophila*, *Bacillus megaterium*, *Pseudomonas aeruginosa*, *Acidovorax sp.*, *Cupriavidus sp.*, *Brevundimonas diminuta*, and others. The table provides valuable insights into the structural diversity and distribution of these enzymes across different bacterial species.

PDB Code	MEROPS ID	Protein	PDB structure group	SP	Lenght
1AI4	S45.001	penicillin G acylase precursor	A	E. coli	763
1AI5	S45.001	penicillin G acylase precursor	A	E. coli	763
1AI6	S45.001	penicillin G acylase precursor	A	E. coli	752
1AI7	S45.001	penicillin G acylase precursor	A	E. coli	763
1AJN	S45.001	penicillin G acylase precursor	A	E. coli	763
1AJP	S45.001	penicillin G acylase precursor	A	E. coli	763
1AJQ	S45.001	penicillin G acylase precursor	A	E. coli	763
1CP9	S45.001	penicillin G acylase precursor	A	Providencia rettgeri	749
1E3A	S45.001	penicillin G acylase precursor	A	E. coli	818
1FXH	S45.001	penicillin G acylase precursor	A	E. coli	766
1FXV	S45.001	penicillin G acylase precursor	A	E. coli	763
1GK9	S45.001	penicillin G acylase precursor	A	E. coli	765
1GKF	S45.001	penicillin G acylase precursor	A	E. coli	764
1GM7	S45.001	penicillin G acylase precursor	A	E. coli	763
1GM8	S45.001	penicillin G acylase precursor	A	E. coli	764
1GM9	S45.001	penicillin G acylase precursor	A	E. coli	763
1H2G	S45.001	penicillin G acylase precursor	A	E. coli	762

1JX9	S45.001	penicillin G acylase precursor	A	E. coli	763
1K5Q	S45.001	penicillin G acylase precursor	A	E. coli	763
1K5S	S45.001	penicillin G acylase precursor	A	E. coli	763
1K7D	S45.001	penicillin G acylase precursor	A	E. coli	763
1KEC	S45.001	penicillin G acylase precursor	A	E. coli	763
1PNK	S45.001	penicillin G acylase precursor	A	E. coli	750
1PNL	S45.001	penicillin G acylase precursor	A	E. coli	750
1PNM	S45.001	penicillin G acylase precursor	A	E. coli	750
4PEL	S45.001	penicillin G acylase precursor	A	Kluyvera citrophila	751
4PEM	S45.001*	penicillin G acylase precursor	A	Kluyvera cryocrescens	784
7REO	S45.001*	penicillin G acylase precursor	B	Kluyvera cryocrescens (G-)	755
7REP	S45.001*	penicillin G acylase precursor	B	Kluyvera cryocrescens (G-)	764
3K3W	S45.001	penicillin G acylase precursor	C	Alcaligenes faecalis	747
3ML0	S45.001	penicillin G acylase precursor	C	Alcaligenes faecalis	746
6NVW	S45.001	penicillin G acylase precursor	D	Bacillus megaterium	719
6NVX	S45.001*	penicillin G acylase precursor	D	Bacillus sp. FJAT-27231	730
6NVY	S45.001*	penicillin G acylase precursor	D	Bacillus thermotolerans	727
8BRQ	S45.001*	penicillin G acylase precursor	D	Bacillus sp. FJAT-27231 (G+)	721
8BRR	S45.001*	penicillin G acylase precursor	D	Bacillus sp. FJAT-27231 (G+)	723
8BRS	S45.001*	penicillin G acylase precursor	D	Bacillus sp. FJAT-27231 (G+)	719
8BRT	S45.001*	penicillin G acylase precursor	D	Bacillus sp. FJAT-27231 (G+)	721
4HSR	S45.002*	cephalosporin acylase precursor	F	Pseudomonas	751
4HST	S45.002*	cephalosporin acylase precursor	F	Pseudomonas	751

7EA4	NoMEROPS	D-succinylase	E	Cupriavidus sp. P4-10-C (G-)	762
7EBY	NoMEROPS	D-succinylase	E	Cupriavidus sp. P4-10-C (G-)	761
4YF9	S45.004	acyl-homoserine lactone acylase	G	Acidovorax sp. MR-S7 (G-)	757
4YFA	S45.004	acyl-homoserine lactone acylase	G	Acidovorax sp. MR-S7 (G-)	757
4YFB	S45.004	acyl-homoserine lactone acylase	G	Acidovorax sp. MR-S7 (G-)	758
5C9I	S45.004	acyl-homoserine lactone acylase	G	Acidovorax sp. MR-S7 (G-)	768
2WYB	S45.004	acyl-homoserine lactone acylase	H	Pseudomonas aeruginosa	710
2WYC	S45.004	acyl-homoserine lactone acylase	H	Pseudomonas aeruginosa	710
2WYD	S45.004	acyl-homoserine lactone acylase	H	Pseudomonas aeruginosa	710
2WYE	S45.004	acyl-homoserine lactone acylase	H	Pseudomonas aeruginosa	710
3L91	S45.004	acyl-homoserine lactone acylase	H	Pseudomonas aeruginosa	710
3L94	S45.004	acyl-homoserine lactone acylase	H	Pseudomonas aeruginosa	707
3SRA	S45.004	acyl-homoserine lactone acylase	H	Pseudomonas aeruginosa	709
3SRB	S45.004	acyl-homoserine lactone acylase	H	Pseudomonas aeruginosa	709
3SRC	S45.004	acyl-homoserine lactone acylase	H	Pseudomonas aeruginosa	710
4BTH	S45.004	acyl-homoserine lactone acylase	H	Pseudomonas aeruginosa	710
4K2F	S45.004	acyl-homoserine lactone acylase	H	Pseudomonas aeruginosa	710
4K2G	S45.004	acyl-homoserine lactone acylase	H	Pseudomonas aeruginosa	710
4M1J	S45.004	acyl-homoserine lactone acylase	H	Pseudomonas aeruginosa	713
4WKS	S45.004	acyl-homoserine lactone acylase	H	Pseudomonas aeruginosa	711
4WKT	S45.004	acyl-homoserine lactone acylase	H	Pseudomonas aeruginosa	713
4WKU	S45.004	acyl-homoserine lactone acylase	H	Pseudomonas aeruginosa	712
4WKV	S45.004	acyl-homoserine lactone acylase	H	Pseudomonas aeruginosa	712

5UBK	S45.004	acyl-homoserine lactone acylase	H	Pseudomonas aeruginosa	709
5UBL	S45.004	acyl-homoserine lactone acylase	H	Pseudomonas aeruginosa	718
1FM2	S45.003	cephalosporin C acylase precursor	I	Brevundimonas diminuta	672
1GHD	S45.002	cephalosporin acylase precursor	I	Pseudomonas sp. 130	673
1GK0	S45.002	cephalosporin acylase precursor	I	Pseudomonas sp. SY-77-1	675
1GK1	S45.002	cephalosporin acylase precursor	I	Pseudomonas sp. SY-77-1	675
1JVZ	S45.002	cephalosporin acylase precursor	I	Brevundimonas diminuta	672
1JW0	S45.002	cephalosporin acylase precursor	I	Brevundimonas diminuta	673
1OR0	S45.002	cephalosporin acylase precursor	I	Pseudomonas sp. SY-77-1	674
2ADV	S45.002	cephalosporin acylase precursor	I	Pseudomonas sp. GK16	683
2AE3	S45.002	cephalosporin acylase precursor	I	Pseudomonas sp. GK16	681
2AE4	S45.002	cephalosporin acylase precursor	I	Pseudomonas sp. GK16	682
2AE5	S45.002	cephalosporin acylase precursor	I	Pseudomonas sp. GK16	681
3JTQ	S45.002	cephalosporin acylase precursor	I	Pseudomonas sp. GK16	676
3JTR	S45.002	cephalosporin acylase precursor	I	Pseudomonas sp. GK16	680
1KEH	S45.003	cephalosporin C acylase precursor	J	Brevundimonas diminuta	683
3S8R	S45.002	cephalosporin acylase precursor	J	Pseudomonas sp. SY-77-1	684
4E55	S45.002	cephalosporin acylase precursor	J	Pseudomonas sp. 130	665
4E56	S45.002	cephalosporin acylase precursor	J	Pseudomonas sp. 130	664
4E57	S45.002	cephalosporin acylase precursor	J	Pseudomonas sp. 130	677

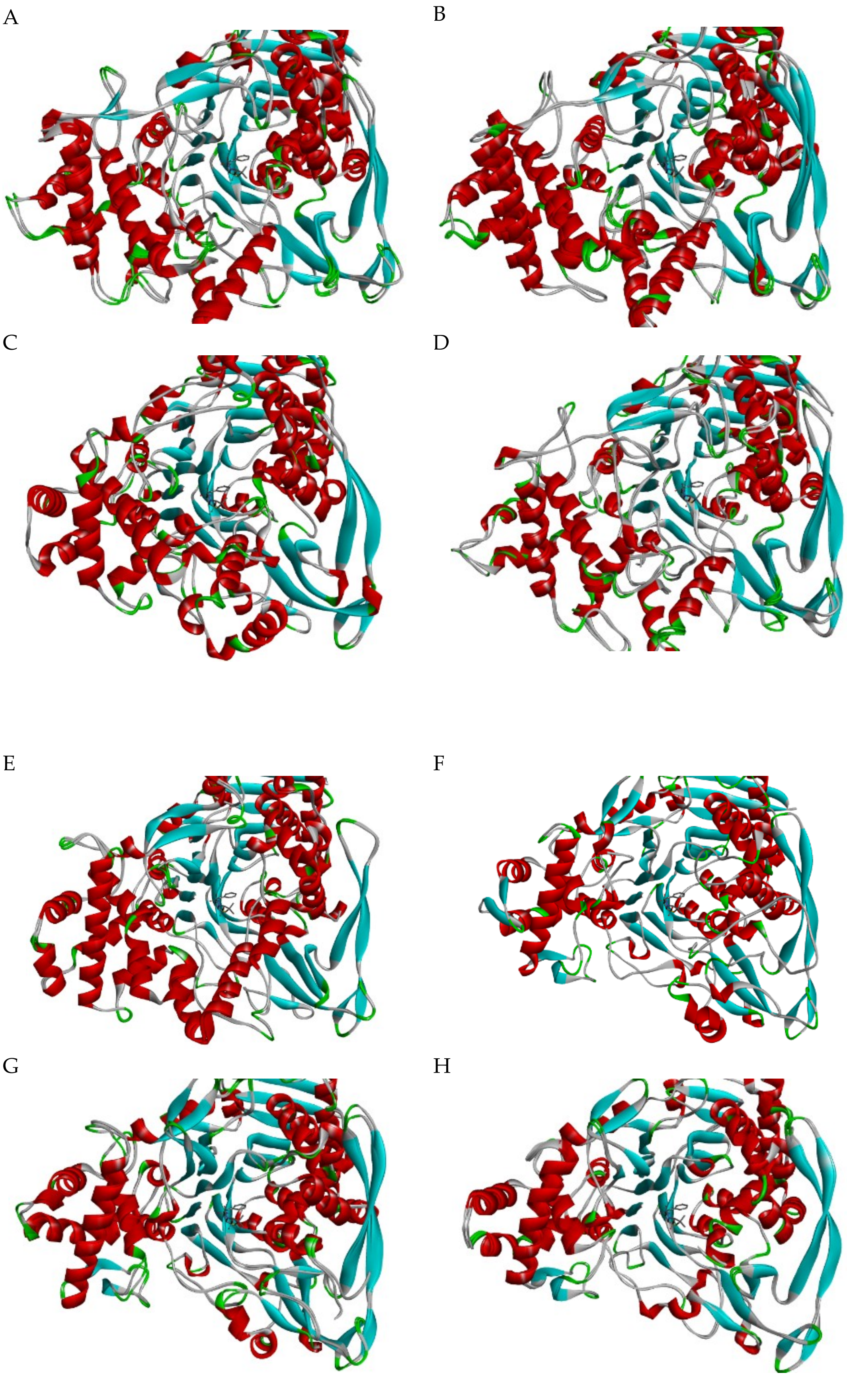
A
B
C
D
E
F
G
H

I

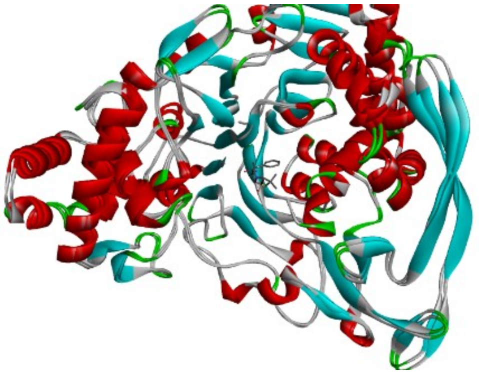

Figure S1. Structural alignment of N-terminal nucleophile (Ntn) amide-hydrolases. (**A**) The structural alignment of proteins exhibiting the highest RMSD within the *E. coli*, *Providencia rettgeri* and *Kluyvera sp.* penicillin G acylase group reported in the Protein Data Bank (PDB) as chain A and chain B separately, specifically referencing entries 1AI4 and 1CP9. (**B**) The structural alignment of proteins exhibiting the highest RMSD within the *Kluyvera cryocrescens* penicillin G acylase group reported in the Protein Data Bank (PDB) as only one chain and with the portion corresponding to the A chain at the end of the amino acid sequence, specifically referencing entries 7REO and 7REP. (**C**) The structural alignment of proteins exhibiting the highest RMSD within the *Alcaligenes faecalis* penicillin G acylase group reported in the Protein Data Bank (PDB) as chain A and chain B separately, specifically referencing entries 3K3W and 3ML0. (**D**) The structural alignment of proteins exhibiting the highest RMSD within the Bacillus sp. penicillin G acylase group reported in the Protein Data Bank (PDB) as chain A and chain B separately, specifically referencing entries 6NVW and 8BRR. (**E**) The structural alignment of proteins exhibiting the highest RMSD within *the Cupriavidus sp.* D-succinylase group reported in the Protein Data Bank (PDB) as only one chain, specifically referencing entries 7EA4 and 7EBY. (**F**) The structural alignment of proteins exhibiting the highest RMSD within the *Pseudomonas sp.* cephalosporin acylase group reported in the Protein Data Bank (PDB) as chain A and chain B separately, specifically referencing entries 4HSR and 4HST. (**G**) The structural alignment of proteins exhibiting the highest RMSD within the *Acidovorax sp.* acyl-homoserine lactone acylase G group reported in the Protein Data Bank (PDB), specifically referencing entries 4YF9 and 5C9I. (**H**) The structural alignment of proteins exhibiting the highest RMSD within the *Pseudomonas aeruginosa* acyl-homoserine lactone acylase group reported in the Protein Data Bank (PDB), specifically referencing entries 3SRA and 5UBK. (**I**) The structural alignment of proteins exhibiting the highest RMSD within the *Brevundimonas diminuta* and *Pseudomonas sp.* cephalosporin acylase group reported in the Protein Data Bank (PDB) as chain A and chain B separately, specifically referencing entries 1GK1 and 1OR0.

Table S2. Su Summary of the identified Structural Conserved Regions (SCRs) within the protein, detailing their locations in consensus numbering and observed structural characteristics. Each SCR is described with its corresponding location and structural features, such as alpha helices, beta sheets, loops, and turns.mmary of the identified SCRs.

SCR	Location in consensus numbering	Observations
1A	52 – 102 A	It starts with a beta hairpin motif, followed by a V-shaped alpha helix (HA), and ends with a short helix (HA'). Key features include conserved Gly residues and specific triplet sequences that cause V-shaped alpha helix.
2A	123 – 131 A	9-amino acid alpha helix (HB).

3A	153 – 172 A	It is a 20-amino-acid long alpha helix (HC) positioned beneath HA, forming the protein's base.
4A	195 – 204 A	It consists of a loop running antiparallel to HC and a perpendicular alpha helix (HD) up to 15 amino acids long, with only the initial 6 amino acids of HD included.
1B	7 – 13 and 21 – 28 B	It begins with a conserved beta sheet (B1-1) with an initial serine crucial for activity, followed by NXWXXG. B1-1 has about 7 amino acids, interrupted by G, A, or R, and followed by a variable region. It continues with an antiparallel beta sheet (B1-2), mirroring B1-1, also with 7 amino acids, ending with a highly conserved proline (P28) as part of the LLANDHPH consensus sequence.
2B	39 – 46 and 50 – 57 B	It starts with an 8-amino acid beta sheet (B2-1) featuring a consistent HL or LH pattern near the C-terminal end, followed by a semi-conserved loop of 2 to 3 amino acids. This is succeeded by an 8-amino acid antiparallel beta sheet (B2-2) with a conserved Gly in the middle.
3B	66 – 91 B	It comprises a beta hairpin motif with a 3-amino acid beta sheet (B2-3), a 3-amino acid coil, and a 6-amino acid beta sheet (B2-4). It features a 6-amino acid coil from the active site and an 8-amino acid beta sheet (B3-1). An alanine, critical for protein activity, is identified in this region. Preceding this amino acid in all sequences is a threonine whose function remains unreported, indicating its potential importance in protein function.
4B	99 – 116 B	It presents a short 4-amino acid beta sheet (B4-1), succeeded by a turn, and a second 4-amino acid beta sheet (B4-2) disrupted by a coiled distortion, typically involving a single amino acid, connecting to the 7-amino acid beta sheet (B5-1).
5B	129 – 143 B	It comprises a hairpin motif consisting of a 9-amino acid beta sheet (B5-2), followed by a turn, and a 4-amino acid beta sheet (B5-3).
6B	162 – 167 B	It is a single beta sheet (B3-2)
7B	194 – 201 B	It is an 8-amino acid alpha helix (H1)
8B	212 – 235 B	It features a 7-amino acid beta sheet (B2-5) at its beginning, followed by a semi-conserved 4 or 5-amino acid turn, and then the beta sheet (B2-6). An interesting pattern is observed in B2-5, with a hydrophobic amino acid in crystallographic structures or a conserved Q in PGA bacterial sequences, followed by a highly conserved N. Although its function is unreported, the presence

		of this N, coupled with its interaction via hydrogen bonding with another highly conserved amino acid (B2-4 T). Like SCR3, SCR8 extends into a coil and the beta sheet (B6-1).
9B	263 – 266 B	In a short beta sheet containing 4 amino acids, the Gly at the extreme C-terminal is highly conserved.
10B	283 – 294 B	This region spans 12 amino acids, consisting of 4 amino acids from B6-2, 3 from the 3_{10} helix, the long-sidechain connector amino acid, and 4 amino acids forming B2-7.
11B	300 – 306 B	It is a beta sheet B (B1-3) and initial segment of a turn in the C-terminal of B1-3 with an N parto of the active site
12B	349 – 357 B	It is a 9-amino acid α-helix (H2), which may potentially extend up to 15 amino acids, though only 9 amino acids fall within an SCR. The consistent segment of H2 commences with a highly conserved R.
13B	375 – 382 B	It is an 8-amino acid alpha helix (H3)
14B	700 - 707	It is an 8-amino acid beta sheet (B1-4)
15B	719 – 722 B	It is an 4-amino acid beta sheet (B1-5)
16B	745 - 765 B	It is delineated by the presence of the 7-amino acid H4, connected through 2 amino acids to the 4-amino acid B1-6, which is then linked by 2 amino acids to the 6-amino acid H5

A

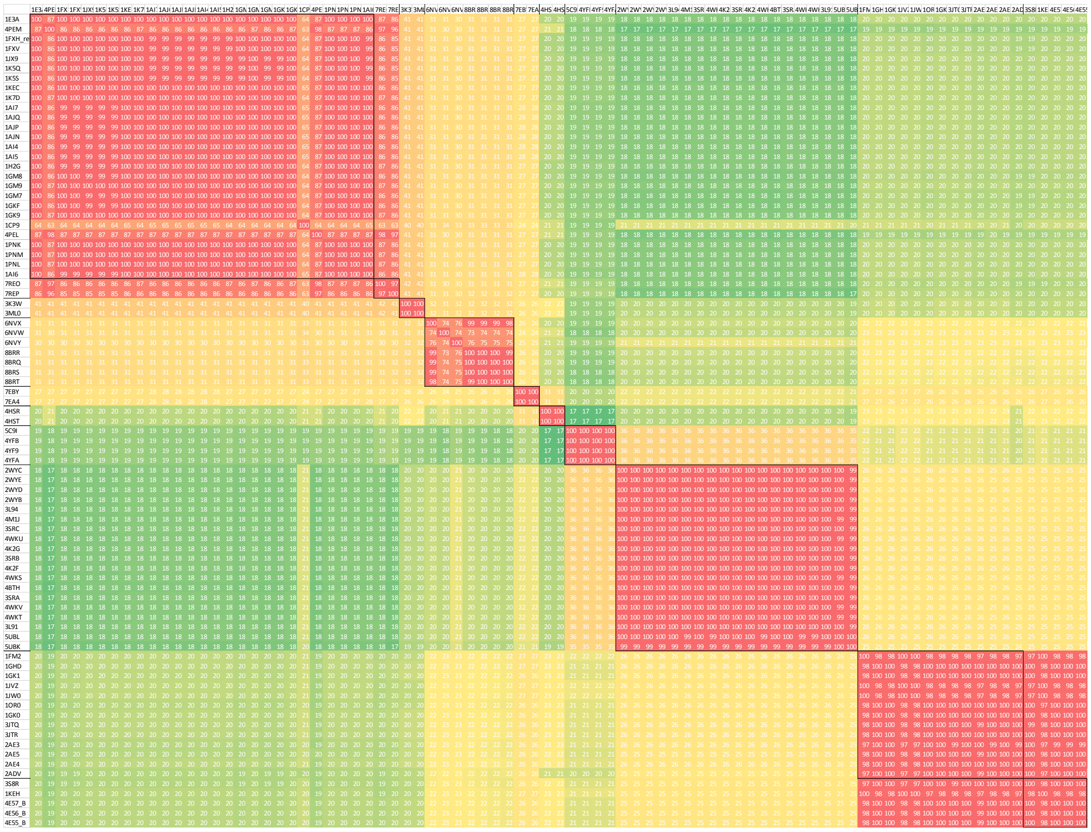

B

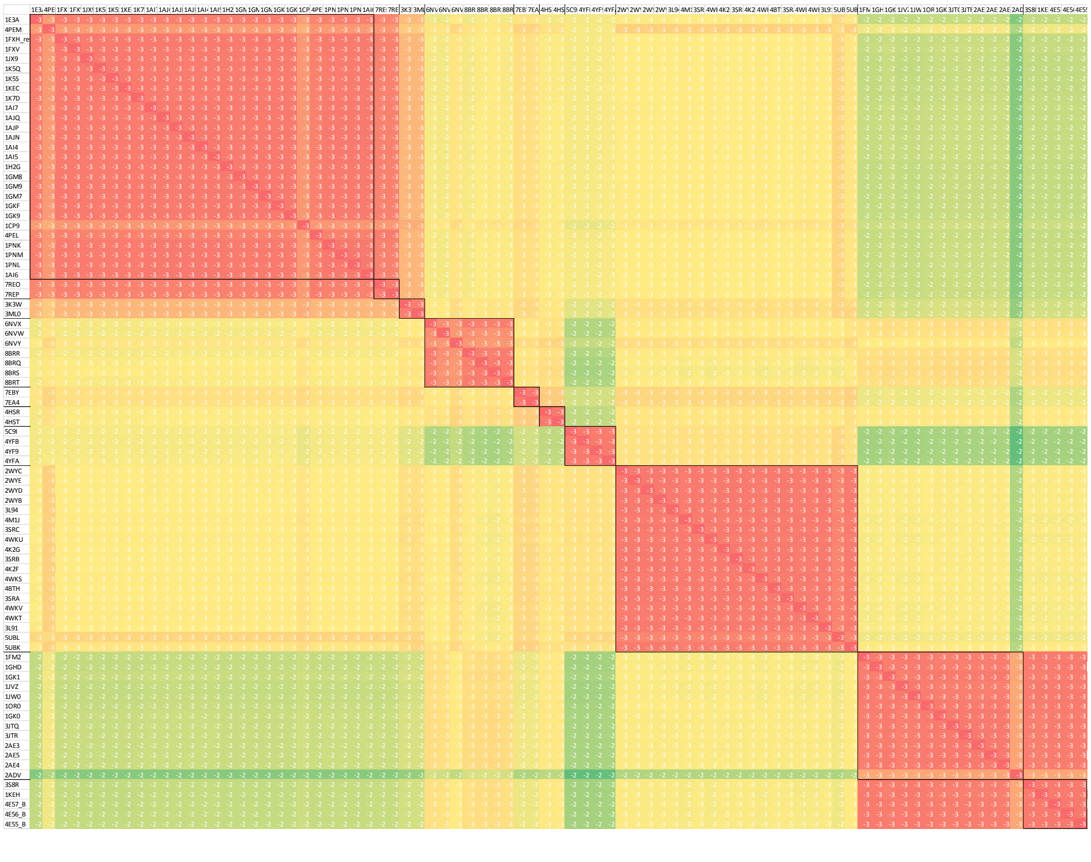

Figure S2. Graphic representation of the sequence and structural identity matrices for the studied crystallographic structures. Red indicates the most similar values, while green denotes the greatest differences in sequence (A) and strucure (B). The PDB estructures in both matrix are ordered in relation wuth Table S1. The boxes delineate the groups in which the structures were clustered; being A the first up-left and continuing in descending order to the right until the lower right box corresponding to group J. The percentage of identity was calculated using the Clustal Omega EMBL server, while the structure similarity score was determined via the SALIGN server. A value of -3 represents the highest possible score among a group of crystallographic structures; specifically, comparing a structure against itself yields a value of -3.

Table S3. The positions corresponding to amino acids within the active sites in structures 1FXH, 1FM2, 1KEH, 2WYB, 3K3W, 4HSR, 4YF9, 6NVW, 7EA4, and 7REO. In bold letter is shown the residues in direct interaction with penicillin in 1FXV.

1FXH	1FM2	1KEH	2WYB	3K3W	4HSR	4YF9	6NVW	7EA4	7REO	Observations
A-M142	A-M145	A-M145	A-L146	A-M143	A-G162	A-N160	A-M145	A-G181	A-M697	In proximity to SCR4A, positioned towards the C-terminal end, this region is noted for its reported interaction with the substrate at the active site [1].
A-R145	A-L148	A-L148	A-E149	A-R146	A-L164	A-A162	A-Y147	No fit	A-R700	It plays a significant role in binding the β-lactam moiety of penicillin G, extending into the solvent and maintaining a distance of 8 Å from the ligand's carboxyl group [2].
A-F146	A-Y149	A-Y149	A-G150	A-F147	A-M165	A-G163	A-F148	A-E182	A-F701	Conserved across various Pseudomonas species but not consistently observed among crystal structures. It can potentially be relocated to facilitate the entry of penicillin to the active site [2].
**B-S1**	**B-S170**	**B-A170**	**B-S1**	**B-S1**	**B-S1**	**B-S1**	**B-S1**	**B-S282**	**B-S1**	At the beginning of SCR1B. Altamente conservada. Aminoácido catalítico. Comprobado en E. Coli [3]. 1KEH, 3S8R, 4E55, 4E56, 4E57 and 5 UBK presented Ala purposely mutated for experimental purposes. This mutation has been reported that the protein completely lost intramolecular autoproteolytic activity. 2AE5 and 4PEL present C. 4PEM present G [4–6]
B-P22	B-P191	B-P191	B-P22	B-P22	B-P22	B-P22	B-P22	B-P303	B-P22	Remarkably conserved residues that interrupt the structural continuity between beta strands 1 and 2 are situated towards the terminus of SCR1B.
**B-Q23**	**B-H192**	**B-H192**	**B-H23**	**B-Q23**	**B-H23**	**B-H23**	**B-Q23**	**B-H304**	**B-Q23**	In the variable region following B1-2, a conserved motif is observed among penicillin G acylase (PGA) genes from Pseudomonas species but not within the crystal structures. This motif constitutes part of the catalytic triad, comprised of Asp, His, and Ser residues, and functions through a hydrogen bond network pivotal for enzyme activity. The protonation of His enhances the electron density in Ser's hydroxyl group, thereby facilitating its nucleophilic attack. His further interacts with Ser1's Oγ and coordinates with the scissile amide bond of penicillin G, playing a crucial role in the formation of the acylenzyme intermediate [3].
B-F24	B-L193	B-L193	B-F24	B-F24	B-R24	B-W24	B-V24	B-R305	B-F24	In the variable region following B1-2, there is an interaction with residue R145 and with penicillin within the active site. Mutations in this residue may influence the enzyme's Km. The presence of leucine (L) is more frequent than phenylalanine [2,7].
B-V56	B-R226	B-R226	B-N57	B-L56	B-P56	B-Q57	B-M56	B-S337	B-L56	Not conserved. Located at the initiation of SCR3B but not within SCR3B itself [7]

B-F57	B-F227	B-F227	B-I58	B-F57	B-F57	B-I58	B-F57	B-I338	B-F57	Not conserved; it can be substituted by hydrophobic residues such as I, V, L, M, A, and F. Located at the initiation of SCR3B, it interacts with penicillin in the active site, and mutations in this residue may impact the enzyme's Km [7].
B-T68	B-T238	B-T238	B-T69	B-T69	B-T69	B-T69	B-T68	B-T349	B-T68	Highly conserved across all species and crystallographic structures, situated proximal to the C-terminal end of B2-4 within SCR3B. Its deduction stems from empirical observation and has not been previously documented.
**B-A69**	**B-V239**	**B-V239**	**B-V70**	**B-A70**	**B-H70**	**B-V70**	**B-A69**	**B-R350**	**B-A69**	Not preserved and consistently located before threonine, this site demonstrates high conservation. Documented as catalytic amino acids in the literature [3].
B-F71	B-G241	B-G241	B-T72	B-P72	B-F72	B-T72	B-Y71	B-Y352	B-A71	Not conserved, situated at the N-terminal end of the coil linking B2-4 and B3-1 within SCR3B [1].
B-W154	B-Y322	B-Y322	B-W162	B-W154	B-L154	B-W165	B-Y158	B-S436	B-W154	Situated within a variable region spanning SCR6B and SCR7B, this residue, V, remains conserved among Streptomyces species. Variations may occur, with possible substitutions to A or I, and within crystallographic structures, W, L, or Y have been observed [2].
B-I177	B-F346	B-F346	B-V187	B-I177	B-H178	B-V190	B-L181	B-E460	B-I177	Conserved across Pseudomonas species but not consistently observed in crystallographic structures, this residue resides at the N-terminus of B2-5, marking the onset of SCR8B [7].
B-N178	B-N347	B-N347	B-N189	B-N178	B-N179	B-N191	B-N182	B-N461	B-N178	This residue, found at the N-terminal end of B2-5 within SCRB8, exhibits high conservation across species and in crystallographic structures. Its presence was deduced through observation and has not been previously reported. It interacts via hydrogen bonding with the T of B2-4, while the other conserved amino acids have not been documented previously.
**B-A241**	**B-N413**	**B-N413**	**B-N269**	**B-N241**	**B-N242**	**B-N278**	**B-N245**	**B-N523**	**B-N241**	Within SCR11B, situated at the initial segment of the turn in the C-terminal of B1-3, this residue demonstrates high conservation. Ala is present exclusively in 1FXV, 1FXH, 1GKF, and 1GM7. To gain structural insights into the nucleophile binding site, they engineered the inactive βN241A variant [2,8]. 5UBK has Asn, for reasons yet unknown as stated in an upcoming publication, representing a catalytic amino acid [3].
**B-R263**	**B-R443**	**B-R443**	**B-R297**	**B-R261**	**B-R263**	**B-R308**	**B-R266**	**B-R547**	**B-R263**	SCR12, located at the terminus of H2, exhibits high conservation across all species. It serves as a catalytic amino acid, as demonstrated in E. coli, coordinating the α-amino group of Ser1 and the carbonyl group of the β-lactam ring [3].

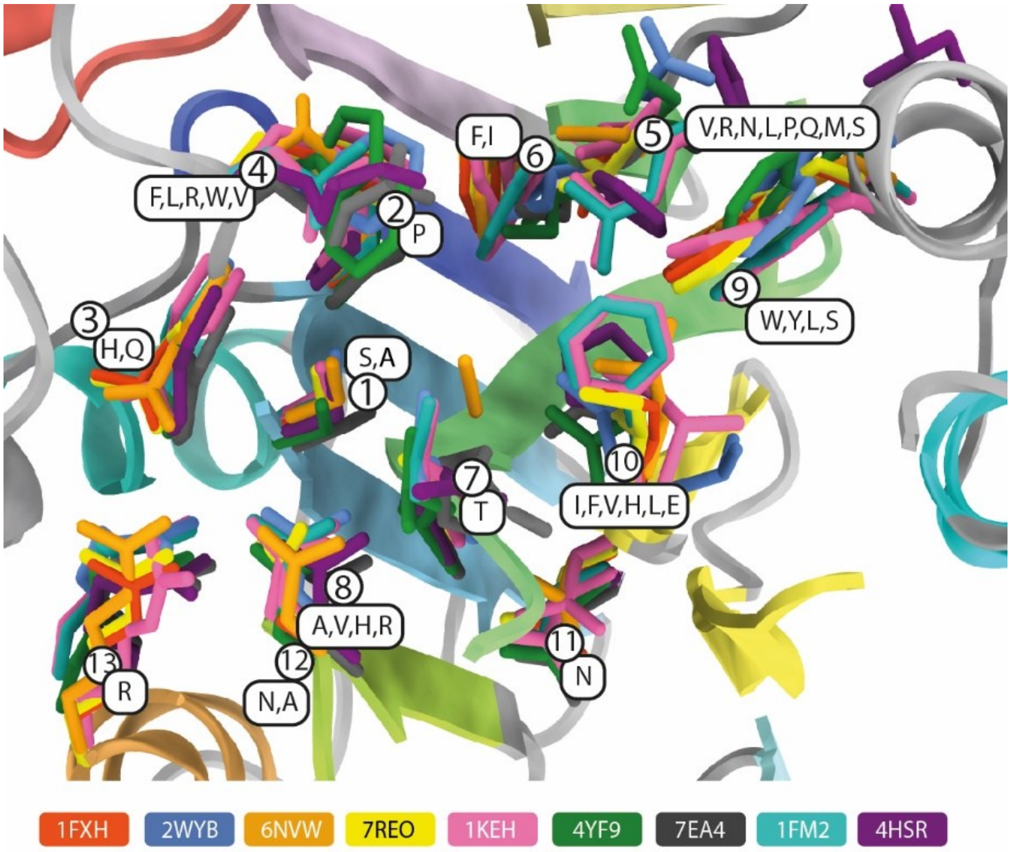

Figure S3. Active sie of N-terminal nucleophile (Ntn) amide-hydrolases. Comparison of the active site in red *E. coli* penicillin G acylase [PDB: 1FXH], in blue *Pseudomonas aeruginosa* acyl-homoserine lactone acylase [PDB: 2WYB], in orange *Bacillus megaterium* penicillin G acylase [PDB: 6NVW], in yellow *Kluyvera cryocrescens* penicillin G acylase [PDB: 7REO], in pink *Brevundimonas diminuta* cephalosporin C acylase [PDB: 1KEH], in green *Acidovorax sp*. MR-S7 acyl-homoserine lactone acylase [PDB: 4YF9], in black *Cupriavidus sp*. P4-10-C D-succinylase [PDB: 7EA4], in cyan *Brevundimonas diminuta* cephalosporin C acylase [PDB: 1FM2], in violet Pseudomonas cephalosporin acylase [PDB: 4HSR].

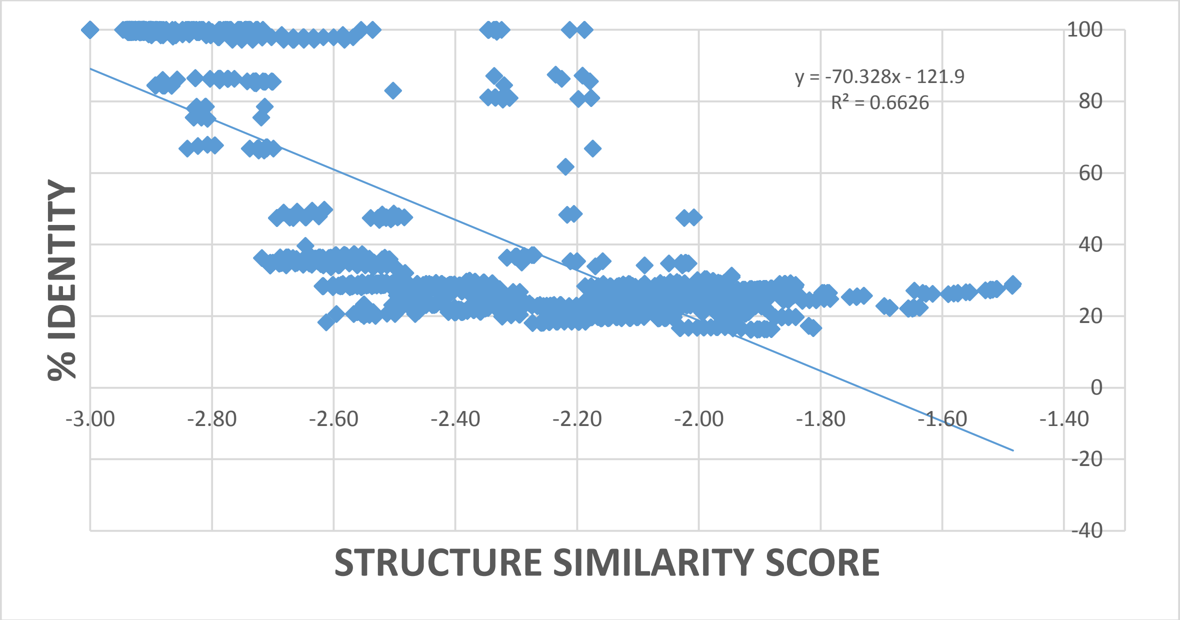

Figure S4. Graph of percentage correlation between sequence identity and structure similarity score. The percentage of identity was calculated using the Clustal Omega EMBL server, while the structure similarity score was determined via the SALIGN server. The graph illustrates a correlation between the two variables (R^2 values are provided in the image).

References

1. Aghajari, N.; Roth, M.; Haser, R. Crystallographic Evidence of a Transglycosylation Reaction: Ternary Complexes of a Psychrophilic α-Amylase. *Biochemistry* **2002**, *41*, 4273–4280, doi:doi.org/10.1021/bi0160516.
2. Alkema, W.B.L.; Hensgens, C.M.H.; Kroezinga, E.H.; de Vries, E.; Floris, R.; van der Laan, J.-M.; Dijkstra, B.W.; Janssen, D.B. Characterization of the β-Lactam Binding Site of Penicillin Acylase of Escherichia Coli by Structural and Site-Directed Mutagenesis Studies. *Protein Eng* **2000**, *13*, 857–863, doi:10.1093/protein/13.12.857.
3. Grigorenko, B.L.; Khrenova, M.G.; Nilov, D.K.; Nemukhin, A. V; Svedas, V.K. Catalytic Cycle of Penicillin Acylase from Escherichia Coli: QM/MM Modeling of Chemical Transformations in the Enzyme Active Site upon Penicillin G Hydrolysis. *ACS Catal* **2014**, *4*, 2521–2529, doi:doi.org/10.1021/cs5002898.
4. Kim, Y.; Kim, S.; Earnest, T.N.; Hol, W.G.J. Precursor Structure of Cephalosporin Acylase: Insights into Autoproteolytic Activation in a New N-Terminal Hydrolase Family. *Journal of Biological Chemistry* **2002**, *277*, 2823–2829, doi:10.1100/tsw.2002.54.
5. Kim, J.K.; Yang, I.S.; Shin, H.J.; Cho, K.J.; Ryu, E.K.; Kim, S.H.; Park, S.S.; Kim, K.H. Insight into Autoproteolytic Activation from the Structure of Cephalosporin Acylase: A Protein with Two Proteolytic Chemistries. *Proceedings of the National Academy of Sciences* **2006**, *103*, 1732–1737, doi:10.1073/pnas.0507862103.
6. Kim, J.K.; Yang, I.S.; Rhee, S.; Dauter, Z.; Lee, Y.S.; Park, S.S.; Kim, K.H. Crystal Structures of Glutaryl 7-Aminocephalosporanic Acid Acylase: Insight into Autoproteolytic Activation. *Biochemistry* **2003**, *42*, 4084–4093, doi:10.1021/bi027181x.

7. Alkema, W.B.L.; Dijkhuis, A.-J.; De Vries, E.; Janssen, D.B. The Role of Hydrophobic Active-Site Residues in Substrate Specificity and Acyl Transfer Activity of Penicillin Acylase. *Eur J Biochem* **2002**, *269*, 2093–2100, doi:10.1046/j.1432-1033.2002.02857.x.
8. McVey, C.E.; Walsh, M.A.; Dodson, G.G.; Wilson, K.S.; Brannigan, J.A. Crystal Structures of Penicillin Acylase Enzyme-Substrate Complexes: Structural Insights into the Catalytic Mechanism. *J Mol Biol* **2001**, *313*, 139–150, doi:10.2210/pdb1gm9/pdb.
